
JOURNAL INFORMATION
==============================
NLM Title Abbreviation: ScientificWorldJournal
Journal ID: ScientificWorldJournal
NLM Journal ID: 101131163
Journal ID: TSWJ

The Scientific World Journal

ISSN: 2356-6140
EISSN: 1537-744X
Publisher: Wiley

ARTICLE INFORMATION
==============================
PMCID: PMC5763808
PMID: 20623092
DOI: 10.1100/tsw.2010.140
Article ID: 594292
Article version: 1
Subjects: Clinical Image

Complete Atelectasis of the Left Lung from Anaplastic Large-Cell Lymphoma

Zhang Liling
Wu Gang *
Cancer Center, Union Hospital, Tongji Medical College, Huazhong University of Science and Technology, Wuhan, China
*Gang Wu: wg1893@hotmail.comAcademic Editor: Daniel Batlle

Publication date: 2010
Electronic publication date: 2010 Jul 6
Volume: 10
First page: 1332
Last page: 1333
Received 2010 May 12; Revised 2010 Jun 14; Accepted 2010 Jun 15
Copyright: Copyright © 2010 Liling Zhang and Gang Wu.
Copyright year: 2010
License: This is an open access article distributed under the Creative Commons Attribution License, which permits unrestricted use, distribution, and reproduction in any medium, provided the original work is properly cited.
License URL: https://creativecommons.org/licenses/by/3.0/

Keywords: pulmonary atelectasis, anaplastic large-cell lymphoma, computed tomography, radiotherapy

==============================
Clinical Image
*TheScientificWorldJOURNAL* (2010) 10, 1332–1333
ISSN 1537-744X; DOI 10.1100/tsw.2010.140

TheScientificWorldJOURNAL
www.thescientificworld.com

### Complete Atelectasis of the Left Lung from Anaplastic Large-Cell Lymphoma

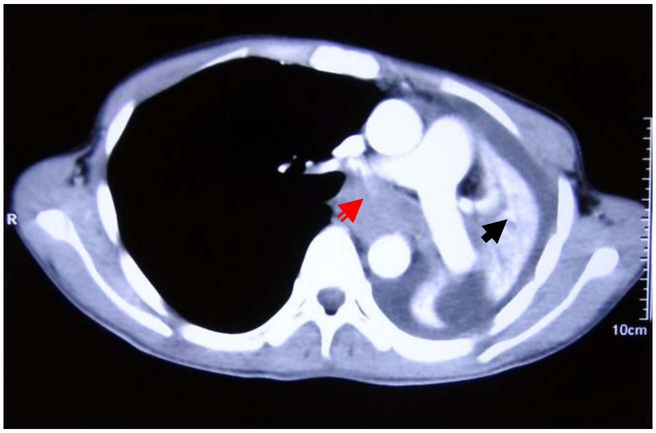

**FIGURE 1.** A contrast-enhanced CT scan shows a mass (red arrow) in the middle mediastinum compressing the left main bronchus, resulting in a complete atelectasis of the left lung (black arrow) and a mediastinal shift toward the left. A pleural effusion is visualized.

Liling Zhang and Gang Wu*

*Cancer Center, Union Hospital, Tongji Medical College, Huazhong University of Science and Technology, Wuhan, China*

**E-mail**: wg1893@hotmail.com

*Received May 12, 2010; Revised June 14, 2010; Accepted June 15, 2010; Published July 6, 2010*

**KEYWORDS**: pulmonary atelectasis, anaplastic large-cell lymphoma, computed tomography, radiotherapy

Acute, complete, pulmonary atelectasis due to intrinsic or extrinsic bronchial obstruction could result in severe dyspnea. An emergency medical intervention should be done promptly on the basis of an accurate diagnosis.

The images we present here are from an 18-year-old boy with severe dyspnea, palpitation, dry cough, and fever for 4 days. Oxygen saturation measured by a pulse oximetry was 80%. Physical examination revealed a 3- × 3-cm firm mass in his right neck. Chest computed tomography (CT) demonstrated a mass (5.4 ×3.5 ×5 cm) in the middle mediastinum compressing the left main bronchus, resulting in a complete atelectasis of the left lung and a mediastinal shift toward the left (Fig. 1). A left pleural effusion was also presented. A biopsy diagnosed an anaplastic lymphoma kinase (ALK)-positive anaplastic large-cell

*Corresponding author.
©2010 with author.
Published by TheScientificWorld; www.thescientificworld.com lymphoma (ALCL) and confirmed the CT findings. A palliative radiotherapy of total 15 Gy in five fractions was delivered from the 1st day of administration. The symptoms were totally resolved after 3 days of radiotherapy, and oxygen saturation level returned to normal. Subsequent chest CT showed the mass had almost completely disappeared with re-expansion of the left lung and return of the mediastinum to the midline, as well as the disappearance of pleural effusion (Fig. 2). The patient then underwent a combination chemotherapy regimen comprising CHOP (cyclophosphamide, adriamycin, vincristine, prednisone).

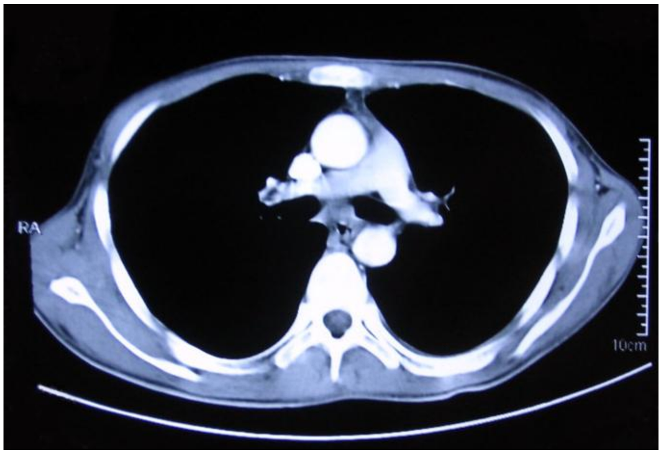

**FIGURE 2**. Chest CT on the 5th day of radiotherapy shows re-expansion of the left lung with and return of the mediastinum to the midline. The mass is nearly completely disappeared.

ALCL involving the mediastinum is not common. Differential diagnosis includes nodular sclerosing Hodgkin’s lymphoma, large B-cell lymphoma, and lymphoblastic lymphoma, which are the three most common types of mediastinal lymphoma[1]. Biopsy and immunohistochemistry are helpful to make a definitive diagnosis. Once histology is known, radio- or chemotherapy could be performed promptly to resolve the complete atelectasis due to extrinsic compressing of the main bronchus by malignant lymphoma[2]. As in reports, cardiopulmonary bypass, Nd:YAG laser, and temporary airway stenting might also be useful in the management of symptomatic airway stenoses[3,4].

### REFERENCES

1. Duwe, B.V., Sterman, D.H., and Musani, A.L. (2005) Tumors of the mediastinum. *Chest* **128**, 2893–2909.
2. Weidmann, E., Gramatzki, M., Wihelm, M., and Mitrou, P.S. (2004) Diagnosis and actual therapy strategies in peripheral T-cell lymphomas: summary of an international meeting. *Ann. Oncol.* **15**, 369–374.
3. Schmidt, B., Massenkeil, G., John, M., Arnold, R., and Witt, C. (1999) Temporary tracheobronchial stenting in malignant lymphoma. *Ann. Thorac. Surg.* **67**, 1448–1450.
4. Shimokawa, S., Watanabe, S., and Niwatsukino, H. (2000) Endobronchial infiltration of malignant lymphoma. *Ann. Thorac. Surg.* **69**, 1985.

**This article should be cited as follows:**

Zhang, L. and Wu, G. (2010) Complete atelectasis of the left lung from anaplastic large-cell lymphoma. *TheScientificWorldJOURNAL* **10**, 1332–1333. DOI 10.1100/tsw.2010.140.
